# Heat Stress Metrics for US Census Tracts 1998–2020

**DOI:** 10.1038/s41597-026-06909-w

**Published:** 2026-02-25

**Authors:** Rouzbeh Rahai, Qinqin Kong, Timur Dogan, Gary W. Evans, Nancy M. Wells

**Affiliations:** 1 Department of Human Centered Design, College of Human Ecology, Cornell University, Ithaca, NY USA; 2 Woods Institute for the Environment, Stanford University, Stanford, CA USA; 3 Department of Architecture, College of Architecture, Art, and Planning, Cornell University, Ithaca, NY USA; 4 Department of Psychology, College of Human Ecology, Cornell University, Ithaca, NY USA

**Keywords:** Climate and Earth system modelling, Risk factors, Climate-change adaptation

## Abstract

Extreme heat exposure is a growing public health threat. Heat-health research has commonly used dry-bulb temperature to characterize heat exposure, partly due to limited availability of spatially explicit, public-health-aligned datasets that integrate multiple meteorological factors to quantify heat stress. We address this gap by providing hourly Heat Index (HI), Wet-Bulb Globe Temperature (WBGT), and Universal Thermal Climate Index (UTCI) for U.S. census tract boundaries across the contiguous United States from 1998–2020. Heat-stress fields were generated by integrating PRISM, ERA5-Land, and National Solar Radiation Database (NSRDB) products, with near-surface temperature and moisture fields reconstructed and ancillary variables interpolated to a harmonized 800-m grid. Heat-stress indices were computed using validated physical models and aggregated to census tracts using area- and population-weighted methods. Validation against station networks shows stable performance for sample year 2010 May–September, with air-temperature root mean squared error (RMSE) of 1.70 °C, Heat Index RMSE of 3.20 °C, WBGT RMSE of 2.90 °C, and UTCI RMSE of 3.26 °C. These tract-level hourly heat-stress datasets enable direct linkage with public health data.

## Introduction

### Background & Summary

Extreme heat exposure is a public health threat that has increased in frequency, duration, and intensity globally^1–3^. In the United States, 21,518 heat-related deaths were recorded between 1999 and 2023, reflecting a 117% increase over that period^4^. Most epidemiologic research to date has relied on dry-bulb temperature as the primary indicator of heat exposure, in part because it is widely available and straightforward to integrate across datasets^5–7^. However, human thermoregulation is influenced by humidity, solar radiation, and wind speed, in addition to air temperature, and these interacting factors determine the physiological burden of heat stress^8,9^. In response, epidemiological research is increasingly adopting Heat Index (HI) and more comprehensive indices such as Wet-Bulb Globe Temperature (WBGT) and Universal Thermal Climate Index (UTCI)^10–12^, which account for multiple meteorological inputs and can outperform dry-bulb temperature in predicting heat-related health outcomes^13,14^. However, progress is constrained by the paucity of heat-stress datasets that (i) compute physically valid heat stress indices at high spatiotemporal resolution, and that (ii) align outputs with public-health geographies.

Heat-stress metrics capture thermal burden across different climatic conditions and vary in formulation, data requirements, and underlying assumptions. Heat Index (HI) estimates perceived temperature using dry bulb temperature and relative humidity and reflects reduced evaporative cooling under humid conditions^15^, which has made it convenient for routine use and public heat warnings^16^. However, HI assumes shading conditions and minimal wind and is less representative under direct solar exposure or substantial air movement^17^. Wet-Bulb Globe Temperature (WBGT) integrates air temperature, humidity, solar radiation, wind speed, and pressure to represent combined radiative and convective heat loads outdoors^18^, and is widely used in occupational and military settings^19,20^. At the same time, WBGT is challenging to estimate at scale because it requires specialized measurements or physically based models to derive wet-bulb and globe temperatures^21^. Universal Thermal Climate Index (UTCI) is a thermal stress indicator incorporating the effects of temperature, humidity, wind, and radiation based on a human thermoregulation model^22,23^. However, UTCI depends on radiative inputs that are often unavailable at high spatial resolution in reanalysis datasets^24,25^, and estimation based on assumed or constant radiation values may introduce additional uncertainty^26,27^. Observational research has found that no single metric is superior to others in assessing population-level heat-related health impacts^28^, which is another motivation to integrate datasets that provide HI, WBGT, and UTCI from the same inputs and in parallel to support robust comparison and reproducible inferences across studies.

Recent data expansion work has increased the availability of gridded heat-stress datasets, but important limitations remain in metric resolution, completeness, and reusability. At the highest spatial detail, some global heat-exposure products provide daily fields at ~0.05° resolution, whereas others offer hourly estimates at coarser spatial scales (typically ~0.1°–0.25°) derived from reanalysis inputs^29,30^. Other datasets can achieve comparable spatial resolution but frequently report only daily summary statistics (e.g., mean, minimum, maximum), and long-horizon global products commonly provide aggregate-hourly heat-stress variables at ~0.25° resolution^31,32^. Many gridded products focus on a single index or a single summary statistic rather than providing parallel estimates of multiple heat-stress metrics derived from the same underlying meteorology. In particular, WBGT is frequently reported using reduced or proxy formulations, such as estimating WBGT from heat index, or computed using “simplified WBGT” in which globe temperature is replaced by dry-bulb temperature due to the absence of radiative terms^29,31,33^. More physically explicit WBGT formulations that resolve natural wet-bulb and globe temperatures require multiple meteorological inputs and iterative calculations, making them computationally expensive to implement at large spatial and temporal scales, leading many global products to rely on approximations^21,34^. Universal Thermal Climate Index (UTCI) remains comparatively less available as a gridded exposure product at fine spatiotemporal resolutions (~0.05° or greater) due to expensive computational and data requirements^30,31^. Finally, reusability is often limited by archived heat-stress datasets that can reach multi-terabyte scales in specialized formats (e.g., NetCDF or GeoTIFF), which may require high-performance computing or specialized geospatial workflows^29,30,32^. It remains uncommon for an analytical gridded dataset to provide HI, WBGT, and UTCI simultaneously, computed at hourly resolution from a harmonized set of meteorological inputs using consistent modeling assumptions, which limits cross-metric comparison and reproducibility across heat-health studies.

Another barrier to heat-health research is the limited availability of exposure data products that are aligned with public-health geographies. Many health surveillance systems and cohort studies restrict access to personally identifiable geographic locations and thus require environmental exposures to be linked at administrative units such as county or census tract boundaries^35–37^. In response, several environmental archives provide tract- or county-level climate summaries, but these products focus on dry-bulb temperature rather than heat-stress indices that incorporate humidity, radiation, and wind^38,39^. Other resources either summarize climate at specific geographies or provide station-level observations, which can lead to geographic gaps in heat-stress availability^40,41^. Addressing these data gaps typically requires intensive preprocessing and spatial aggregation to translate fine-resolution gridded exposures to census or county boundaries, which often entails substantial data storage and computational demands that increase analyst burden. A recent county-level database of population-weighted daily heat metrics (including HI, WBGT, and UTCI) helps bridge this gap for county-based analyses^42^, but comparable tract-level heat-stress products remain unavailable because they require meteorological reanalysis across far more spatial detail.

To improve public health research capacity and facilitate reproducibility, we provide hourly, area-weighted and population-weighted averages of HI, WBGT, and UTCI for U.S. Census tract boundaries across the contiguous United States from 1998–2020. These data will enable linking with health outcomes and sociodemographic variables on specific days and points in time, and help facilitate analysis of how heat exposure can contribute to morbidity and mortality through environmental factors such as unequal access to cooling, greenspace, or housing quality, which are delineated at census tract levels^38,43^. To our knowledge, this is the first dataset to provide comprehensive historic hourly heat stress estimates across the contiguous United States at high resolution, aggregated to census tract geographies, using a fully validated, physically based modeling framework.

## Methods

### Workflow overview

**Fig. 1:**
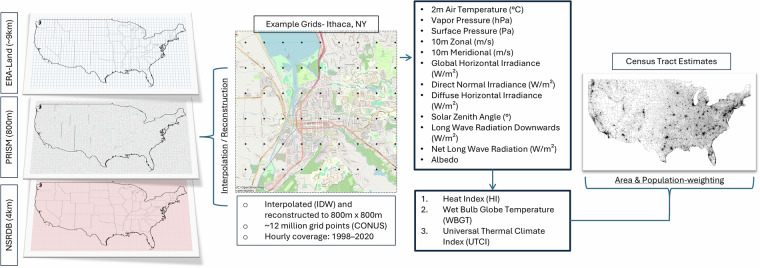
shows modeling workflow.

We provide hourly, area-weighted and population-weighted averages of Heat Index (HI), Wet-Bulb Globe Temperature (WBGT), and Universal Thermal Climate Index (UTCI) within census tract boundaries across the contiguous United States (CONUS) from 1998–2020. Heat-stress values are derived from PRISM climate data (800 m)^44^, ERA5-Land (~9 km)^45^, and the National Solar Radiation Database (NSRDB) (4 km)^46^. We combine the diurnal profile from ERA5-Land and the daily extreme or mean constraints from PRISM to develop hourly temperature and vapor pressure on the PRISM grid, and other NSRDB and ERA5-Land variables were spatially interpolated to the common 800 m grid used by PRISM. Heat-stress indices are calculated at each 800 m grid cell and then spatially aggregated to census tracts. Figure 1 presents an overview of the modeling workflow.

### Data acquisition

We acquired high-resolution meteorological and solar data for the contiguous United States from the PRISM climate group, ERA5-Land, and the National Solar Radiation Database (NSRDB). PRISM is a high-resolution gridded climate dataset developed by the PRISM Climate Group at Oregon State University, based on station observations and physiographically informed interpolation^24^. PRISM provides daily near-surface meteorological fields for the contiguous United States at 800 m (30-arc-second) resolution. From each PRISM grid cell, we extracted daily minimum and maximum air temperature (${{\rm{T}}}_{{\rm{a}},\min }$, ${{\rm{T}}}_{{\rm{a}},\max }$), and daily mean dew-point temperature (${{\rm{T}}}_{{\rm{d}},{\rm{mean}}}$) for the 1998–2020 period^44^. ERA5-Land is a global reanalysis product developed by the European Centre for Medium-Range Weather Forecasts (ECMWF) that provides multi-variable hourly meteorological fields at 0.1° × 0.1° (~9 km) resolution^47^. From each ERA5-Land grid cell, we extracted 2 m air temperature (${{\rm{T}}}_{{\rm{a}}})$, dewpoint temperature (${{\rm{T}}}_{{\rm{d}}})$, surface pressure (${{\rm{P}}}_{{\rm{s}}})$, 10 m zonal (*u*) and meridional (*v*) wind components, surface long-wave radiation downwards ${({\rm{L}}}^{\downarrow })$ and surface net long-wave radiation (${{\rm{L}}}^{* })$ at hourly interval for the same 1998–2020 period^48^. NSRDB v3 provides 30-min solar radiation fields on a 4 km grid from 1998 to 2020^49^. From each NSRDB grid cell we extracted global horizontal irradiance (GHI), direct normal irradiance (DNI), diffuse horizontal irradiance (DHI), solar zenith angle (${\theta }_{z}$), and albedo $({\rm{\alpha }}$) for daily, 30-minute intervals between 1998–2020^50^. Variables from all sources were extracted at their native temporal resolutions—30-min (NSRDB), hourly (ERA5-Land), and daily (PRISM)—and aligned to a common hourly PRISM-day framework in UTC (24 hourly values per PRISM day, running from 12:00 UTC the day before to 11:00 UTC on the PRISM-labeled day). We stored intermediate fields as gridded Parquet time-series on the PRISM 800-m grid for downstream heat-stress and radiation calculations and for subsequent spatial aggregation. Table 1 describes each original raw variable acquired from each reanalysis product.

**Table 1:** Original Input Variables.

Parameter	Symbol	Units	Spatial Resolution	Temporal Resolution	Source
Mean Near-surface Dew point Temperature	${{\rm{T}}}_{{\rm{d}},{\rm{mean}}}$	$^\circ$C	800 m	Daily	PRISM
Maximum Near-surface Temperature	${{\rm{T}}}_{{\rm{a}},\max }$	$^\circ$C	800 m	Daily	PRISM
Minimum Near-surface Temperature	${{\rm{T}}}_{{\rm{a}},\min }$	$^\circ$C	800 m	Daily	PRISM
2 m Air Temperature	${{\rm{T}}}_{{\rm{a}}}$	${\rm{K}}$	0.1° × 0.1° (~9 km)	Hourly	ERA5-Land
Surface Pressure	${{\rm{P}}}_{{\rm{s}}}$	Pa	0.1° × 0.1° (~9 km)	Hourly	ERA5-Land
10 m Zonal Wind	*u*	$\frac{{\rm{m}}}{{\rm{s}}}$	0.1° × 0.1° (~9 km)	Hourly	ERA5-Land
10 m Meridional Wind	*v*	$\frac{{\rm{m}}}{{\rm{s}}}$	0.1° × 0.1° (~9 km)	Hourly	ERA5-Land
2 m Dew Point Temperature	${{\rm{T}}}_{{\rm{d}}}$	${\rm{K}}$	0.1° × 0.1° (~9 km)	Hourly	ERA5-Land
Surface long-wave radiation downwards	${{\rm{L}}}^{\downarrow }$	$\frac{{\rm{J}}}{{{\rm{m}}}^{2}}$	0.1° × 0.1° (~9 km)	Hourly	ERA5-Land
Surface net long-wave radiation	${{\rm{L}}}^{* }$	$\frac{{\rm{J}}}{{{\rm{m}}}^{2}}$	0.1° × 0.1° (~9 km)	Hourly	ERA5-Land
Global Horizontal Irradiance	GHI	$\frac{{\rm{W}}}{{{\rm{m}}}^{2}}$	4 km	Half-hourly	NSRDB
Direct Normal Irradiance	DNI	$\frac{{\rm{W}}}{{{\rm{m}}}^{2}}$	4 km	Half-hourly	NSRDB
Diffuse Horizontal Irradiance	DHI	$\frac{{\rm{W}}}{{{\rm{m}}}^{2}}$	4 km	Half-hourly	NSRDB
Solar Zenith Angle	${{\rm{\theta }}}_{{\rm{z}}}$	Degrees (°)	4 km	Half-hourly	NSRDB
Albedo	${\rm{\alpha }}$	Unitless (0–1)	4 km	Half-hourly; updates every 8 days	NSRDB

### Preprocessing

The PRISM 800 m grid was adopted as the reference grid for all interpolation and reconstruction steps. Since ERA5-Land provides hourly accumulated downwelling longwave radiation (${{\rm{L}}}^{\downarrow })$ and hourly accumulated net longwave radiation (${{\rm{L}}}^{* })$ expressed as J/m², these accumulations were converted to hourly longwave fluxes (W/m²) by differencing successive hourly accumulation fields and dividing by the one-hour accumulation interval (3600 s). NSRDB variables are provided as 30-minute time series and were aggregated to hourly resolution by averaging adjacent half-hour values for global horizontal irradiance (GHI), direct normal irradiance (DNI), diffuse horizontal irradiance (DHI), solar zenith angle (${\theta }_{z}$), and surface albedo ($\alpha$). Temporal aggregation of solar zenith angle was computed from the hourly mean of $\cos {\theta }_{z}$ and converted back to degrees to preserve radiative consistency. Albedo is provided as an 8-day product and therefore did not vary within the hourly aggregation.

### Reconstruction

Near-surface air temperature and moisture explain the majority of variance in heat-stress indices, including Heat Index (HI), Wet-Bulb Globe Temperature (WBGT), and Universal Thermal Climate Index (UTCI). We reconstructed hourly near-surface temperature and moisture fields on the PRISM 800 m grid using constrained temporal rescaling and disaggregation methods^51,52^. ERA5-Land hourly data were used to define the intraday shape of diurnal variability, while PRISM daily fields provided spatially detailed constraints on daily minimum and maximum temperature, and mean moisture conditions. Because PRISM supplies station-informed daily information at high spatial resolution but lacks sub-daily variability, and ERA5-Land provides physically consistent hourly variability at coarser resolution, ERA5-Land (∼9 km) was used to supply the reference diurnal profile, while PRISM (800 m) conserved the target daily temperature extremes and mean moisture state. ERA5-Land hourly values were aligned to PRISM days by matching the PRISM 24-hour window. Aligning to PRISM days ensures that reconstructed diurnal profiles remain consistent with PRISM daily temperature extrema and means. Each PRISM grid cell was linked to its nearest ERA5-Land grid cell to extract the local diurnal profile used for reconstruction. This approach preserves PRISM daily constraints at a fine resolution while borrowing intraday structure from a physically consistent reference dataset, consistent with established temporal disaggregation methods^51,52^. ERA5-Land temperature and dew point were converted to Celsius prior to reconstruction.

#### Temperature reconstruction

Hourly near-surface air temperature was reconstructed by combining ERA5-Land diurnal variability with PRISM daily temperature constraints over the PRISM-day hourly window. For each ERA5-Land grid cell, we first constructed a dimensionless diurnal profile by normalizing hourly 2 m air temperature to the ERA5-Land daily extrema:

$$f(h)=\frac{{{\rm{T}}}_{{\rm{a}},{\rm{E}}{\rm{R}}{\rm{A}}5-{\rm{L}}{\rm{a}}{\rm{n}}{\rm{d}}}\,({\rm{h}})-\,{{{\rm{T}}}_{{\rm{a}},{\rm{E}}{\rm{R}}{\rm{A}}5-{\rm{L}}{\rm{a}}{\rm{n}}{\rm{d}}}}_{min}}{{{{\rm{T}}}_{{\rm{a}},{\rm{E}}{\rm{R}}{\rm{A}}5-{\rm{L}}{\rm{a}}{\rm{n}}{\rm{d}}}}_{max}\,-\,{{{\rm{T}}}_{{\rm{a}},{\rm{E}}{\rm{R}}{\rm{A}}5-{\rm{L}}{\rm{a}}{\rm{n}}{\rm{d}}}}_{min}};f(h)\in [0,1].$$

where $f(h)$ is the unitless diurnal scaling function, $h=0,\ldots ,23$ indexes the PRISM-day hourly window, ${{\rm{T}}}_{{\rm{a}},{\rm{E}}{\rm{R}}{\rm{A}}5-{\rm{L}}{\rm{a}}{\rm{n}}{\rm{d}}}({\rm{h}})$ is ERA5-Land hourly 2 m air temperature, and ${{{\rm{T}}}_{{\rm{a}},{\rm{E}}{\rm{R}}{\rm{A}}5-{\rm{L}}{\rm{a}}{\rm{n}}{\rm{d}}}}_{min}$ and ${{{\rm{T}}}_{{\rm{a}},{\rm{E}}{\rm{R}}{\rm{A}}-{\rm{L}}{\rm{a}}{\rm{n}}{\rm{d}}}}_{max}$ are the corresponding daily minimum and maximum temperatures. This normalization preserves the timing and shape of the diurnal temperature cycle while removing amplitude information. The ERA5-Land diurnal profile was rescaled to PRISM daily temperature extremes:

$${T}_{a}(h)={T}_{a,{PRISM}\min }+f(h)({T}_{a,{PRISM}\max }-{T}_{a,{PRISM}\min })$$

where ${T}_{a}\left(h\right)$ is the reconstructed hourly air temperature at the PRISM grid cell, and ${T}_{a,{\rm{PRISM}},\min }$ and ${T}_{a,{\rm{PRISM}},\max }$ are PRISM daily minimum and maximum temperatures at 800 m resolution. This constrained rescaling enforces PRISM’s spatially detailed daily temperature bounds while retaining ERA5-Land’s intraday timing and shape structure.

#### Vapor pressure reconstruction

Hourly near-surface vapor pressure was reconstructed by combining the intraday moisture structure from ERA5-Land with daily moisture constraints from PRISM over the PRISM-day hourly window. We first aligned hourly 2 m dew point temperature ${T}_{d,{\rm{ERA}}5-{\rm{Land}}}(h)$ with the PRISM-day 24 hour window. A daily mean ERA5-Land dew point temperature ${T}_{d,{\rm{ERA}}5-{\rm{Land}},{\rm{mean}}}$ was then calculated over this window. Thereafter, a dew-point correction was defined as the difference between the PRISM and ERA5-Land daily mean dew point temperatures at the target grid cell:

$$\Delta {T}_{d}={T}_{d,{\rm{PRISM}},{\rm{mean}}}-{T}_{d,{\rm{ERA}}5-{\rm{Land}},{\rm{mean}}}$$

Where $\Delta {T}_{d}$ represents the dew-point correction applied to the ERA5-Land hourly dew point profile. Where ${T}_{d,{\rm{PRISM}},{\rm{mean}}}$ is the PRISM daily mean dew point temperature. The correction was then applied additively to the ERA5-Land hourly dew point profile:

$${T}_{d}(h)={T}_{d,{\rm{ERA}}5-{\rm{Land}}}(h)+\Delta {T}_{d}$$

where ${T}_{d}\left(h\right)$ is the reconstructed hourly dewpoint temperature at the PRISM grid cell. The corrected hourly dew point temperatures were then converted to vapor pressure using the Buck formulation for saturation vapor pressure^53^:

$$e(h)={e}_{s}\left({{\rm{T}}}_{{\rm{d}}}({\rm{h}})\right)$$

where ${e}_{s}$ denotes saturation vapor pressure and $e(h)$ is the reconstructed hourly vapor pressure (hPa). This approach preserves the physically consistent intraday moisture variability from ERA5-Land while enforcing PRISM’s spatially detailed daily mean moisture state in dew-point space. To ensure thermodynamic consistency, reconstructed hourly vapor pressure was constrained to not exceed saturation vapor pressure computed from the reconstructed hourly air temperature.

### IDW Interpolation

We used inverse-distance weighting (IDW)^54^ to resample selected gridded meteorological inputs from ERA5-Land (~9 km) and NSRDB (4 km) onto the PRISM 800 m reference grid. For ERA5-Land, we interpolated hourly surface pressure (sp), 10 m wind components (u10, v10), and longwave flux fields derived from ERA’s accumulated radiation products (${{\rm{L}}}^{\downarrow }$ and ${{\rm{L}}}^{* }$). For NSRDB, we interpolated global horizontal irradiance (GHI), direct normal irradiance (DNI), diffuse horizontal irradiance (DHI), solar zenith angle (θz), and surface albedo (α) after aggregating the native 30-min series to hourly resolution. IDW was implemented as a local, four-neighbor scheme: for each PRISM grid cell center, we identified the four nearest source points using a k-d tree built on projected coordinates, computed weights from planar distances, and formed each hourly value as the weighted mean. Our approach ignores missing neighbor values, such that IDW could reduce to nearest-neighbor weighting when only one valid source point is available. IDW is beneficial in this case because it weights source values by proximity and allows explicit distance cutoffs (we imposed 9 km for ERA5-Land and 4 km for NSRDB), which reduces edge artifacts and prevents unintended long-range influences. This is conceptually appropriate because meteorological and radiative variables often vary continuously over space^55^. Moreover, it protects edge coordinates from needing a fixed four-point grid unlike bilinear interpolation, thus avoiding one missingness problem^56^. This approach has been widely used in environmental and climate data processing, including standard remapping workflows in climate toolchains and spatial interpolation of meteorological fields^55^.

### Ancillary calculations

Prior to computing heat stress metrics, we derived relative humidity and 10-m wind speed at hourly resolution. Relative humidity was calculated from our reconstructed grid hourly air temperature and vapor pressure using the Buck vapor pressure relationship^53^. Horizontal wind speed was computed from the interpolated vector magnitude of the zonal and meridional wind components. We evaluated an elevation-adjusted surface pressure correction based on PRISM–ERA5-Land elevation differences but found no meaningful differences in heat stress outputs relative to directly interpolated surface pressure; thus, we kept our interpolated values for completeness in approach. For elevation adjustment, our approach is described herein: Mean surface elevation for each PRISM grid cell was computed using area-weighted sampling of U.S. Geological Survey 3DEP digital elevation models^57^. Elevation was extracted from ERA5-Land’s time-invariant field database, and a reference grid was constructed that mapped each PRISM cell to its nearest ERA5-Land grid for use in interpolation calculations. Surface pressure was adjusted from the native reanalysis reference elevation to the local grid-cell elevation using the hypsometric equation, assuming hydrostatic balance and temperature-dependent air density, and adjustments were applied over land grid cells only^58,59^:

$$\begin{array}{c}{{\rm{P}}}_{{\rm{s}},{\rm{a}}{\rm{d}}{\rm{j}}{\rm{u}}{\rm{s}}{\rm{t}}{\rm{e}}{\rm{d}}}={{\rm{P}}}_{{\rm{s}},{\rm{r}}{\rm{e}}{\rm{f}}{\rm{e}}{\rm{r}}{\rm{e}}{\rm{n}}{\rm{c}}{\rm{e}}}\,\exp \left(-,\frac{{\rm{g}}({{\rm{z}}}_{{\rm{E}}{\rm{R}}{\rm{A}}5-{\rm{L}}{\rm{a}}{\rm{n}}{\rm{d}}}-{{\rm{z}}}_{{\rm{P}}{\rm{R}}{\rm{I}}{\rm{S}}{\rm{M}}})}{{{\rm{R}}}_{{\rm{d}}}({T}_{\bar{a,PRISM{\rm{m}}{\rm{e}}{\rm{a}}{\rm{n}}}})}\right)\\ {\rm{g}}=9.80665{\rm{m}}{{\rm{s}}}^{-2};{{\rm{R}}}_{{\rm{d}}}=287.05{{\rm{J}}{\rm{k}}{\rm{g}}}^{-1}{{\rm{K}}}^{-1}\end{array}$$

where ${{\rm{P}}}_{{\rm{s}},{\rm{adjusted}}}$ is adjusted surface pressure, and ${{\rm{P}}}_{{\rm{s}},{\rm{reference}}}$ is interpolated surface pressure from the ERA5-Land grid. ${{\rm{z}}}_{{\rm{E}}{\rm{R}}{\rm{A}}5-{\rm{L}}{\rm{a}}{\rm{n}}{\rm{d}}}$ is the original grid elevation and ${{\rm{z}}}_{{\rm{PRISM}}}$ is the reference grid elevation. ${T}_{a,{PRISM}{\rm{mean}}}$ is the reference grid average temperature from PRISM.

### Heat stress calculations

*Heat Index (HI)* estimates perceived temperature using air temperature and relative humidity^15^. We calculated heat index using the MetPy Python package^60^, which implements the Rothfusz regression formula used by the U.S. National Weather Service^16,61^. We computed hourly HI across all 800 m grid points and converted air temperature from Celsius to Fahrenheit to meet MetPy requirements. We did not mask any output based on temperature and humidity constraints and all outputs were returned in degrees Celsius for downstream analysis.

*Wet-Bulb Globe Temperature (WBGT)* combines air temperature, humidity, wind speed, and solar radiation into a single measure of thermal stress^18^. We computed hourly WBGT using *PyWBGT* Python package^62^, which implements the full Liljegren physical model^18^. The Liljegren model simulates energy exchange on wet-bulb and globe sensors and is widely regarded as the most robust method for outdoor WBGT estimation. Outdoor WBGT is estimated as a weighted combination of three simulated temperatures^42,63,64^:

$${WBGT}=0.7{\rm{\cdot }}{Tw}+0.2{\rm{\cdot }}{Tg}+0.1{\rm{\cdot }}{Ta}$$

Where natural wet-bulb temperature (*Tw*) reflects evaporative cooling due to humidity and wind, globe temperature (*Tg*) captures radiant heat from the environment, and dry-bulb temperature (*Ta*) represents the ambient air temperature.

Inputs into the *PyWBGT* model included hourly 2 m air temperature (${T}_{a}$), relative humidity ($\mathrm{RH}$), surface pressure (*Ps*), 10-m wind speed (${U}_{10m}$), global horizontal irradiance ($\mathrm{GHI}$), diffuse horizontal irradiance ($\mathrm{DHI}$), and solar zenith angle (${\theta }_{z}$) at each 800 m grid cell. We converted air temperature from degrees Celsius to Kelvin for the Liljegren formulation and while our input was 10 m wind speed, the model internally converts to 2 m level. We clipped relative humidity to the 0–100 percent range. We derived the direct beam fraction as ${f}_{\mathrm{dir}}=1-\mathrm{DHI}/\mathrm{GHI}$ and set ${f}_{\mathrm{dir}}$ = 0 when global horizontal irradiance (GHI) ≤ 1. We converted solar zenith angle (${\theta }_{z})$ to cosine zenith ($\cos {\theta }_{z}$), and for nighttime conditions $\cos {\theta }_{z}$ was replaced with a small negative sentinel value. We then converted model outputs from Kelvin to degrees Celsius for downstream analysis.

*Universal Thermal Climate Index (UTCI)* represents outdoor thermal stress for a reference walking person by combining air temperature, wind speed, humidity, and mean radiant temperature into a single equivalent temperature scale^22,23^. We computed hourly UTCI across all 800 m grid cells using the *Thermofeel* Python package^65^, which implements the sixth order polynomial regression approximation by Brode *et al*. 2012^23^. Inputs to *Thermofeel’s* UTCI calculation included 2-m air temperature (${T}_{a}$), vapor pressure (*e*), 10-m wind speed (${U}_{10m}$), global horizontal irradiance (GHI), direct normal irradiance (DNI), solar zenith angle (${\theta }_{z}$), surface albedo ($\alpha$), and ERA5-Land downwelling (${L}^{\downarrow }$) and net longwave radiation (${L}^{* }$) at each grid cell. We converted air temperature from degrees Celsius to Kelvin. We computed mean radiant temperature (${T}_{{mrt}}$) with Thermofeel from shortwave and longwave radiation by supplying GHI as surface shortwave solar radiation downwards, net shortwave radiation $(1-\alpha )\,{\rm{GHI}}$, DNI as direct solar radiation from the Sun, its horizontal component ${\rm{DNI}}* \cos ({\theta }_{z})$, ERA5-Land downwelling and net longwave radiation, and the cosine of solar zenith angle $\cos ({\theta }_{z})$ truncated at zero; negative shortwave fluxes were set to zero. Thermofeel then evaluated UTCI as a function of ${T}_{a}$, ${U}_{10m}$, ${T}_{{mrt}}$, and $e$ for each hourly timestamp and grid cell, and we converted outputs from Kelvin to degrees Celsius. Following the UTCI operational procedure by Brode *et al*. 2012, we implemented validity limits where input meteorology lay within the validated domain of $-50\le {T}_{a}\le {50}^{\circ }{\rm{C}}$, and $-30\le ({T}_{{mrt}}-{T}_{a})\,\le$ 70°C, RH > 5%, and vapor pressure $e < 50\,{\rm{hPa}}$. While UTCI’s polynomial regression allows calculation at higher wind speeds (30 m/s), we followed the recommendation by Di Napoli and colleagues^26,66^ and restricted valid UTCI values to wind speeds between $0.5{m}/s\le {U}_{10m}\le 17{\rm{m}}/{\rm{s}}$. UTCI values were additionally set to NaN where any required meteorological input was non-finite.

### Census tract estimates

Hourly gridded weather variables at 800 m resolution were spatially aggregated to U.S. Census Tract boundaries using area-weighted and population-weighted mapping between PRISM grid cells and tract boundaries. First, we obtained U.S. Census tract boundary shapefiles from the National Historical Geographic Information System (NHGIS), maintained by the Institute for Social Research and Data Innovation at the University of Minnesota^67^. For each decennial period, we used tract boundaries corresponding to the appropriate census vintage (e.g., 2000 boundaries for 2000–2009 data). A static grid-to-tract lookup table was generated by assigning PRISM grid-cell centroids to census tract polygons. For each census tract polygon, gridded climate values were averaged across assigned PRISM cells using two different weighting schemes. Area-weighted aggregation was performed using latitude-based weights (cosine of latitude) to account for variation in grid-cell area across the Earth. Population-weighted aggregation was performed using annual gridded population estimates from the WorldPop Global dataset for the respective year (where the 2000 WorldPop population surface was used for 1998–1999 due to data availability)^68^. Population counts at 100 m resolution were resampled to the 800 m PRISM grid and used as weights when computing tract-level averages. All points represent centroids of 800 m spatial grids. This approach preserves geographic alignment between climate exposure areas and geographic political boundaries^69,70^. Aggregating gridded climate data to administrative units using area-weighted and population-weighted methods enables estimation of heat exposure in specific boundaries and facilitates linkage to sociodemographic and health datasets to further model population-level effects^42,71,72^.

## Data Records

### Data records and use

We developed an open-access dataset of area-weighted and population-weighted, hourly heat exposure estimates aggregated to U.S. Census Tract boundaries across the contiguous United States. The datasets are stored in parquet format and each file represents two half UTC days and is stored in the following format: *heatstress_tract_area_and_popweighted_[DATE]_[DATE + 1]_popy[Population YEAR]_v[Vintage Year].parquet*, with total storage of all files around 515GB. Specifically, each Parquet file corresponds to a single PRISM day and contains tract-level hourly heat stress estimates spanning a 24-hour UTC exposure window from 12:00 UTC on the previous calendar day to 11:00 UTC on the labeled day. This convention maintains temporal consistency between daily PRISM products and the sub-daily meteorological inputs used in heat stress modeling while avoiding ambiguities associated with calendar-day aggregation. All heat exposure data products are publicly available through Figshare (10.25452/figshare.plus.31040902)^73^. A complete description of the census-tract parquet file structure, including variable names, units, and definitions, is provided in Table 2.

**Table 2:** Data elements and definitions of datasets.

Variable name	Units	Description
GEOID	—	Census tract identifier (NHGIS-compatible)
year	—	Year of observation (UTC-based)
month	—	Month of observation (UTC-based)
day	—	Day of observation (UTC-based)
time	hour	Hour of day (0–23, UTC-based)
temp_C_used_area	°C	Area-weighted tract mean air temperature
temp_C_used_pop	°C	Population-weighted tract mean air temperature
rh_pct_used_area	%	Area-weighted tract mean relative humidity
rh_pct_used_pop	%	Population-weighted tract mean relative humidity
HI_C_area	°C	Area-weighted Heat Index
HI_C_pop	°C	Population-weighted Heat Index
WBGT_C_area	°C	Area-weighted Wet-Bulb Globe Temperature
WBGT_C_pop	°C	Population-weighted Wet-Bulb Globe Temperature
UTCI_C_area	°C	Area-weighted Universal Thermal Climate Index
UTCI_C_pop	°C	Population-weighted Universal Thermal Climate Index

**Fig. 2:**
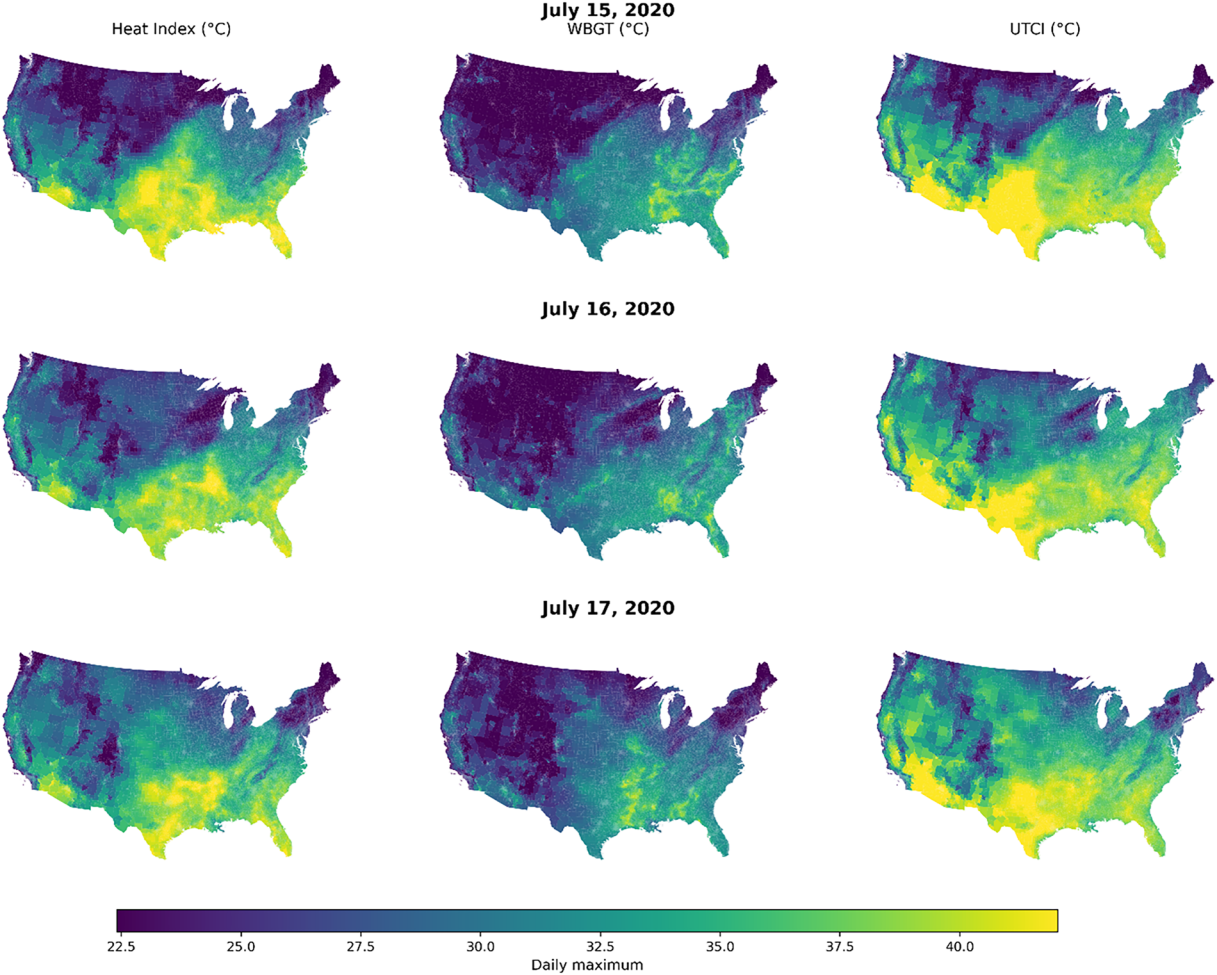
shows example visualization using census tract exposure estimates.

One example of data use is that a public health research analyst may be interested in identifying where peak daily heat stress reaches hazardous levels during a heat event. Figure 2 demonstrates how daily maximum Heat Index, WBGT, and UTCI can be extracted from the hourly census-tract data and mapped to characterize the spatial intensity and geographic extent of area-weighted extreme heat on a given day.

These tract-level heat exposure estimates can be modeled using area-weighted or population-weighted extremes, and then be directly linked to sociodemographic, health, and built-environment indicators at census tract levels, such as those available from the National Neighborhood Data Archive (NaNDA)^38^ which provide harmonized U.S. Census tract data spanning multiple decades. For example, analysts may integrate heat stress maxima with tract-level measures of income, age structure, housing conditions, or health vulnerability to examine inequities in extreme heat exposure, identify populations at elevated risk, and support climate-informed public health planning and intervention targeting.

## Technical Validation

**Fig. 3:**
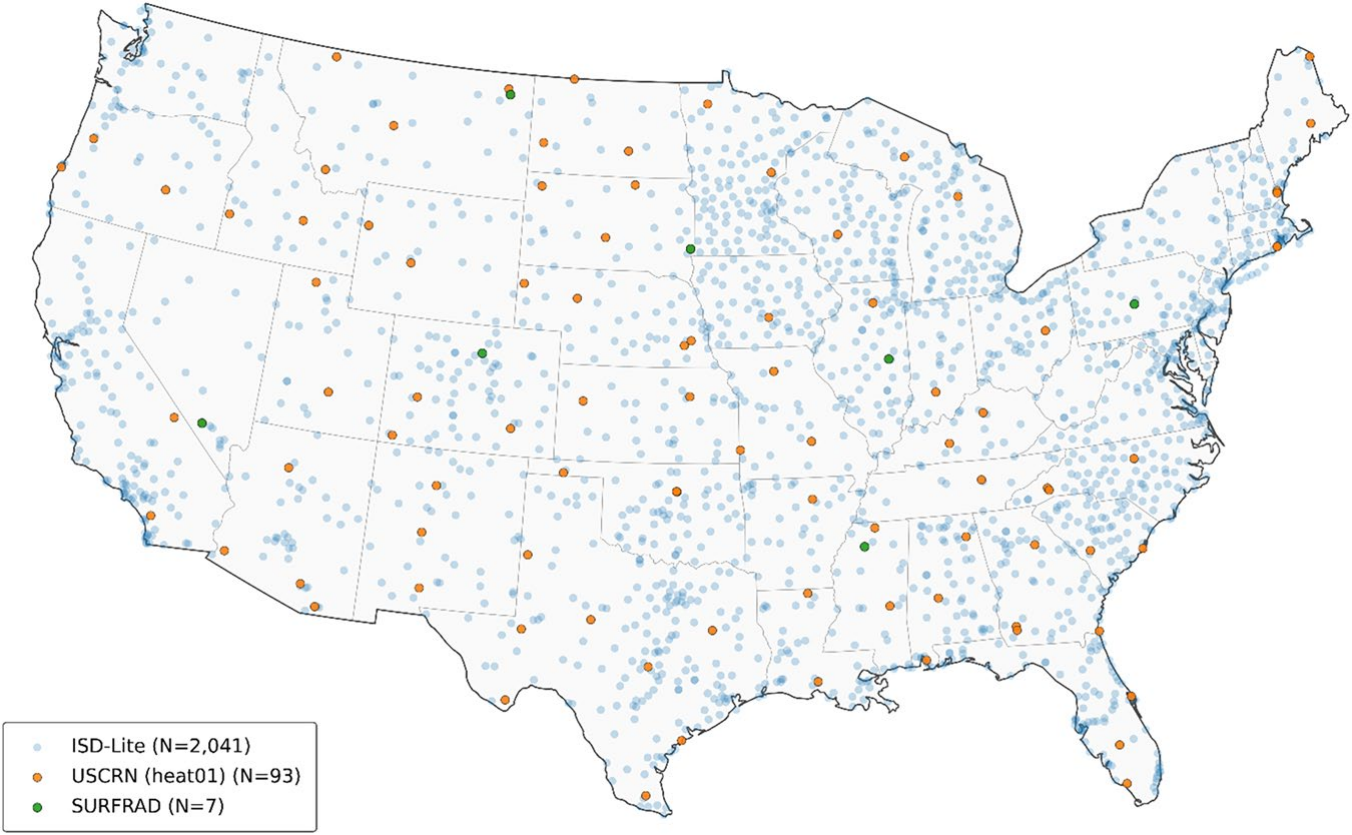
shows total unique stations and locations of stations used to validate heat exposure metrics and their components. Different validation subsample sizes were used due to metric completeness within a larger pool of unique stations.

We conducted technical validation of our reconstructed gridded meteorological fields and derived heat exposure indices used to produce the census-tract exposure products. Validation relied on three independent, station-based observational networks with coverage across the contiguous United States for May–September during 2010. Our gridded air temperature, vapor pressure, and relative humidity fields were evaluated against hourly observations from the NOAA Integrated Surface Database–Lite (ISD-Lite) (N = 2,041)^74^, and parallel comparisons were performed against the parent input grids from ERA5-Land meteorological fields. Derived heat-stress metrics, including Heat Index (HI) and Wet-Bulb Globe Temperature (WBGT), were validated using observations from the U.S. Climate Reference Network (USCRN) heat01 product (N = 93)^75^, while Universal Thermal Climate Index (UTCI) was evaluated using station-derived estimates from the SURFRAD network (N = 7)^76^. Validation was performed at hourly resolution using PRISM-day–aligned 24-hour windows (12:00 UTC to 11:00 UTC), with station locations mapped to the nearest gridded model cell and filtered using distance thresholds consistent with the spatial resolution of each gridded product (800 m for our reconstructed dataset, and 9 km for ERA5-Land). Model performance was summarized using bias and root-mean-square error (RMSE) pooled across the validation window, with window-level estimates weighted by the total number of matched station-hours contributing to each estimate. Figure 3 shows the CONUS locations of stations that contributed observations to the validation analyses across ISD-Lite, USCRN, and SURFRAD sites.

**Fig. 4:**
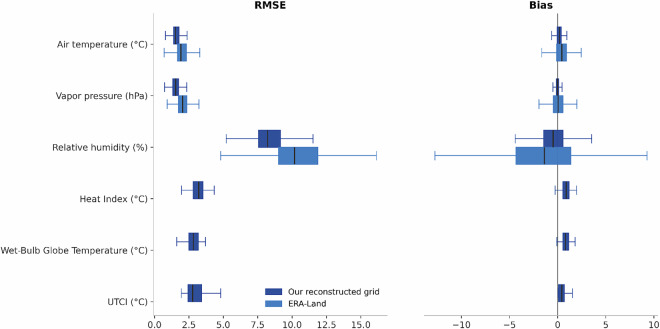
shows pooled RMSE and bias for each variable and product during the May–September 2010 validation period. Boxplots summarize error distributions across all matched station–model comparisons.

**Fig. 5:**
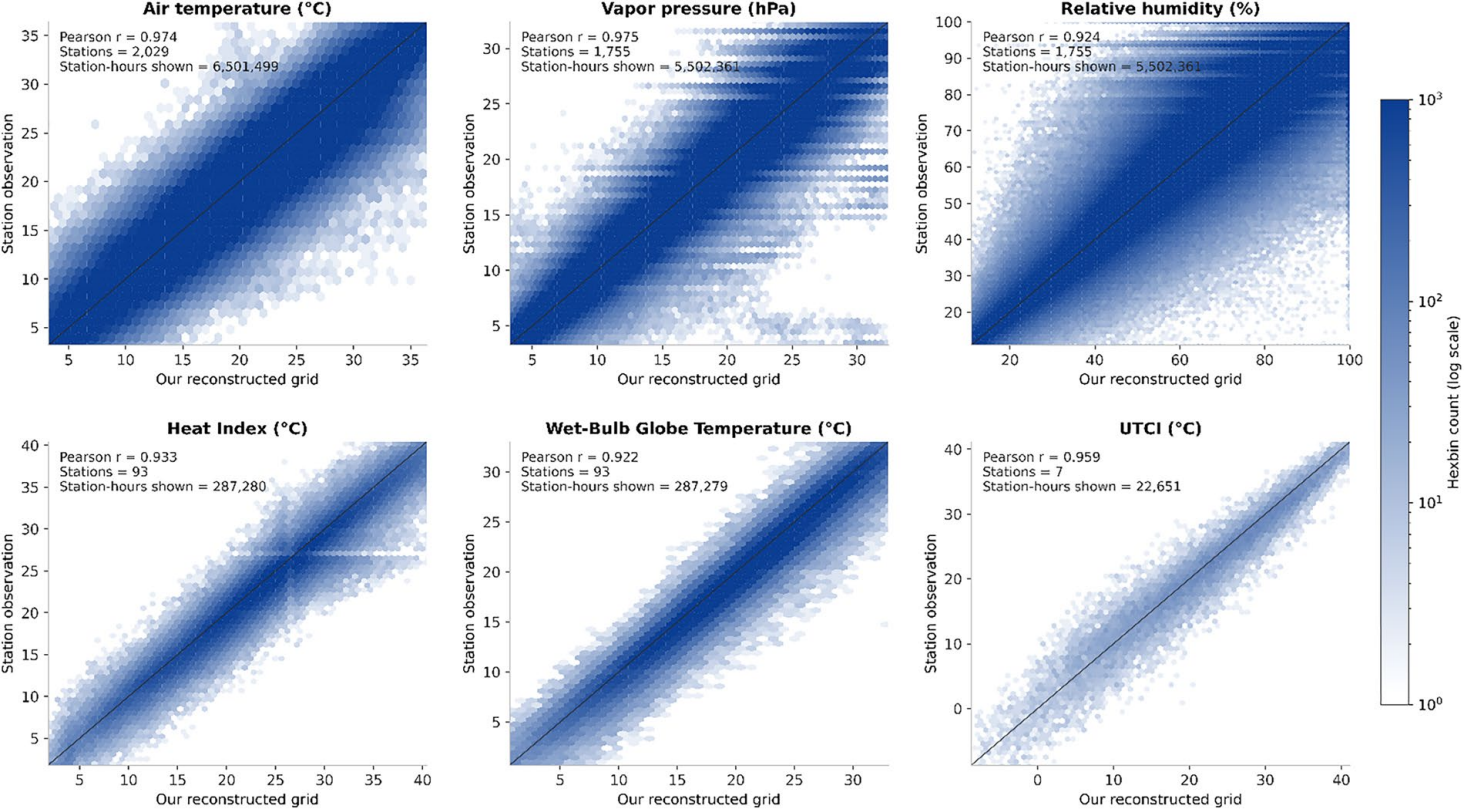
shows station observations versus reconstructed grid values for meteorological variables and heat-stress indices. Hexbin density plots show the distribution of matched station–model pairs; Pearson correlation coefficients are reported in each panel.

Validation was conducted against station observations for a single extended summer period spanning May–September 2010 (Table 3). For our reconstructed grid, air temperature RMSE was 1.70 °C with a mean bias of + 0.17 °C and a correlation of r = 0.97, based on nearest-neighbor station matching within 800 m across 2,029 stations and 6.5 million matched station-hours. Vapor pressure RMSE was 1.70 hPa with a mean bias of −0.02 hPa (r = 0.98), while relative humidity RMSE was 8.89% with a mean bias of −0.40% (r = 0.92). Across all evaluated meteorological variables, the reconstructed grid exhibited lower RMSE and higher correlation than corresponding ERA5-Land inputs evaluated using identical temporal alignment but matched at ERA5-Land’s native 9 km resolution. ERA5-Land air temperature RMSE was 2.17 °C (r = 0.96), vapor pressure RMSE was 2.25 hPa (r = 0.96), and relative humidity RMSE was 11.11% (r = 0.88). Heat-stress indices derived from the reconstructed fields were evaluated against independent observational products. When compared with USCRN heat01 observations, Heat Index RMSE was 3.20 °C with a mean bias of + 0.92 °C (r = 0.93), and WBGT RMSE was 2.90 °C with a mean bias of + 0.84 °C (r = 0.92), based on 93 stations and approximately 287,000 matched hours. UTCI was evaluated separately using station-based UTCI estimates derived from SURFRAD radiation measurements computed with the same Thermofeel Python modeling framework applied to the gridded data. Under this approach, UTCI RMSE was 3.26 °C with a mean bias of + 0.59 °C and a correlation of r = 0.96 across seven SURFRAD stations. Full validation results are reported in Table 3. Figure 4 summarizes the validation results in Table 3, showing pooled RMSE and bias by variable and product for the May–September 2010 period. The boxplots provide comparisons of error magnitudes across datasets. Figure 5 shows how reconstructed values compare directly with station observations. The density plots illustrate the overall agreement across variables and report correlation values across all matched station hours.

**Table 3:** Full validation results.

Product	Reference	Variable	Distance filter	Stations unique (N)	Matched hours (total)	Bias	RMSE	Correlation (r)
ERA5-Land	ISD-Lite	Air temperature (°C)	≤9 km	2027	6,501,576	0.388	2.170	0.957
ERA5-Land	ISD-Lite	Relative humidity (%)	≤9 km	1751	5,495,615	−1.409	11.110	0.879
ERA5-Land	ISD-Lite	Vapor pressure (hPa)	≤9 km	1751	5,495,615	0.013	2.248	0.956
Our reconstructed grid	ISD-Lite	Air temperature (°C)	≤800 m	2029	6,501,499	0.173	1.697	0.974
Our reconstructed grid	ISD-Lite	Relative humidity (%)	≤800 m	1755	5,502,361	−0.402	8.890	0.924
Our reconstructed grid	ISD-Lite	Vapor pressure (hPa)	≤800 m	1755	5,502,361	−0.024	1.699	0.975
Our reconstructed grid	USCRN (heat01)	Heat Index (°C)	≤800 m	93	287,280	0.923	3.202	0.933
Our reconstructed grid	USCRN (heat01)	Wet-Bulb Globe Temperature (°C)	≤800 m	93	287,279	0.843	2.902	0.922
Our reconstructed grid	SURFRAD	UTCI (°C)	≤800 m	7	22,651	0.591	3.263	0.959

RMSE magnitudes reported here are comparable to those reported for other continental-scale gridded meteorological and heat-stress datasets evaluated against station observations^63^, but several limitations should be noted. The reconstructed meteorology relies on inverse-distance weighting of primarily radiation components and does not explicitly incorporate physiographic or land-surface predictors beyond what is already embedded in reanalysis products, which may smooth localized spatial gradients. We also did not conduct a dedicated station-based validation of shortwave or longwave radiation inputs. Because UTCI and to a lesser extent WBGT already combines the effects of radiation, wind, and temperature, we did not perform a separate validation of radiation alone. UTCI was validated using SURFRAD network inputs, which provides high-quality measurements of shortwave and longwave radiation needed to estimate mean radiant temperature, although only a small number of such stations are available (N = 7). Prior studies further indicate that the type of heat-stress metric most strongly associated with heat-related mortality varies across countries and climatic contexts^28^, suggesting that no single index is universally optimal. Accordingly, the inclusion of multiple heat-stress indices is intended to support flexible, context-specific use rather than to prescribe a single measure of heat exposure.

## Data Availability

All heat exposure data products are publicly available through Figshare (10.25452/figshare.plus.31040902)^73^.

## Code availability

Our reconstruction, interpolation, heat-stress calculation, and census-tract aggregation steps are implemented in one end-to-end Python workflow, while the technical validation is implemented in a second Python workflow. Both are publicly available on GitHub: https://github.com/rouzbehrahai/Heat-Stress-Metrics-for-US-Census-Tracts-1998-2020.
